# Brain Functional Topology Alteration in Right Lateral Occipital Cortex Is Associated With Upper Extremity Motor Recovery

**DOI:** 10.3389/fneur.2022.780966

**Published:** 2022-03-03

**Authors:** Qianqian Huang, Dinghong Lin, Shishi Huang, Yungang Cao, Yun Jin, Bo Wu, Linyu Fan, Wenzhan Tu, Lejian Huang, Songhe Jiang

**Affiliations:** ^1^Rehabilitation Medicine Center, The Second Affiliated Hospital of Wenzhou Medical University, Wenzhou, China; ^2^Intelligent Rehabilitation Research Center, China-USA Institute for Acupuncture and Rehabilitation, Wenzhou Medical University, Wenzhou, China; ^3^Department of Neurology, The Second Affiliated Hospital and Yuying Children's Hospital of Wenzhou Medical University, Wenzhou, China; ^4^Department of Information, The Second Affiliated Hospital and Yuying Children's Hospital of Wenzhou Medical University, Wenzhou, China; ^5^Department of Radiology, The Second Affiliated Hospital and Yuying Children's Hospital of Wenzhou Medical University, Wenzhou, China; ^6^Department of Neuroscience, Feinberg School of Medicine, Northwestern University, Chicago, IL, United States

**Keywords:** stroke, upper extremity recovery, resting-state fMRI, right lateral occipital cortex, functional connectivity network

## Abstract

Stroke is a chief cause of sudden brain damage that severely disrupts the whole-brain network. However, the potential mechanisms of motor recovery after stroke are uncertain and the prognosis of poststroke upper extremity recovery is still a challenge. This study investigated the global and local topological properties of the brain functional connectome in patients with subacute ischemic stroke and their associations with the clinical measurements. A total of 57 patients, consisting of 29 left-sided and 28 right-sided stroke patients, and 32 age- and gender-matched healthy controls (HCs) were recruited to undergo a resting-state functional magnetic resonance imaging (rs-fMRI) study; patients were also clinically evaluated with the Upper Extremity Fugl-Meyer Assessment (FMA_UE). The assessment was repeated at 15 weeks to assess upper extremity functional recovery for the patient remaining in the study (12 left- 20 right-sided stroke patients). Global graph topological disruption indices of stroke patients were significantly decreased compared with HCs but these indices were not significantly associated with FMA_UE. In addition, local brain network structure of stroke patients was altered, and the altered regions were dependent on the stroke site. Significant associations between local degree and motor performance and its recovery were observed in the right lateral occipital cortex (R LOC) in the right-sided stroke patients. Our findings suggested that brain functional topologies alterations in R LOC are promising as prognostic biomarkers for right-sided subacute stroke. This cortical area might be a potential target to be further validated for non-invasive brain stimulation treatment to improve poststroke upper extremity recovery.

## Introduction

Stroke, a common medical emergency, remains the main cause of long-term disability and death in the world (1). Usually, stroke patients usually show hemiplegia, especially affecting the upper limb (2). Independence of the patients in activities of daily living depends on the recovery of their upper limb motor functions (3), and accurate prognosis of the recovery would enable realistic goal-setting and guide the allocation of rehabilitation resources. However, the prognosis of poststroke upper extremity recovery is still a challenge.

The most common neuroimaging biomarker to predict poststroke upper extremity recovery is measuring the integrity of the corticospinal tract (4–8). But neurological impairment poststroke can sometimes exceed expectations of stroke severity because stroke not only leads to focal, location-dependent neurological symptoms, but also causes widespread effects in remote hemispheres through functional networks (9–11). Researchers have recently discovered that along with the disorders of structural connectivity (12), reorganization of functional networks is related to the prognosis of patients (13–17). However, at present, there have been few reports on how the reorganization of brain functional networks predicts recovery of clinical motor function (18).

The properties of brain functional networks can be explored using graph theory. Resting-state fMRI (rs-fMRI) combined with network analysis demonstrated that the motor execution networks of convalescent stroke patients shifted to a more random topology (19); the local efficiency and small worldness of stroke patients were significantly higher and lower than those of healthy controls (HCs), respectively, and the latter was increased to a level close to that of the HCs during rehabilitation (20, 21). Moreover, an earlier study has shown functional network topological properties associated with poststroke outcomes in acute ischemic stroke patients (22). Therefore, we hypothesized that the brain functional topological properties were associated with an upper extremity motor recovery after stroke.

In this study, by taking advantage of a modest sample size (57 stroke patients and 32 age- and gender-matched HCs), global and local voxel-based graph theory analyses were performed and the correlation between brain network dysfunction and behavior in subacute stroke was fully investigated. First, after the patients were divided into two different subgroups based on stroke site, global graph properties of the two subgroups, and their association with clinical measurement were assessed independently. Following this, voxel-based degree maps between patients and their matched HCs were compared and association with clinical measurement was evaluated. Finally, using the significantly associated regions from the last step as regions of interest (ROIs), an ROI-based functional connectivity (FC) analysis was performed to explore the significantly different FC regions between patients and the HCs and found an ROI that can evaluate the extent to which upper extremity recovered postrehabilitation.

## Materials and Methods

### Participants

We recruited 62 patients with first-time ischemic stroke between May 2017 and July 2020 at the Department of Physical Medicine and Rehabilitation in the Second Affiliated Hospital of Wenzhou Medical University, China. A total of five patients were removed from further data analyses because they did not pass our data quality control (see Section Quality control for details). To be eligible, participants must have (1) been between 30 and 85 years of age; (2) had their first stroke within the past month; (3) subcortical lesion restricted to the basal ganglia, internal capsule, corona radiate, or brainstem. Participants were excluded if they had the previous history of brain neurosurgery or epilepsy, or any MRI contraindications. The difference in lesion location of ischemic stroke is associated with different functional outcomes (23). Therefore, in this study, the stroke patients were divided into two subgroups: left-sided stroke patients and right-sided stroke patients.

In addition, 32 gender-, age-matched HCs were recruited. HCs were excluded if they (1) were <30 or more than 85 years old; (2) had psychiatric disease; (3) reported history of brain neurosurgical procedures and/or epilepsy; (4) were not suitable for MRI scan. All the stroke patients and HCs were right-handed. The demographical and clinical characteristics of stroke patients and HCs are detailed in Table 1.

**Table 1:** Demographic and clinical characteristics of left-sided and right-sided stroke patients and HCs.

	**Left-sided**		**Right-sided**		**HCs**		**Left-sided**	**Right-sided**	**Left- vs**.
	**(*****n*** **=** **29)**		**(*****n*** **=** **28)**		**(*****n*** **=** **32)**		**vs. HCs *p-*value**	**vs. HCs*p-*value**	**Right-sided *p-*value**
Age, mean (SD), years^a^	62.38	(11.10)	64.71	(8.56)	65.22	(3.07)	0.192	0.770	0.379
Gender, male (%)^b^	19	(65.52)	15	(53.57)	15	(46.88)	0.143	0.605	0.358
Right handedness (%)	29	(100)	28	(100)	32	(100)	1.000	1.000	1.000
Time after stroke, mean (SD), days	15.62	(7.09)	18.18	(8.31)		–	–	–	0.216
Hypertension (%)	22	(75.86)	20	(71.43)		–	–	–	0.704
Diabetes (%)	12	(41.38)	19	(67.86)		–	–	–	0.045
Baseline FMA_UE, mean (SD)	33.79	(23.65)	28.79	(16.62)		–	–	–	0.339
Follow-up FMA_UE, mean (SD)^*^	49.42	(22.52)	46.60	(19.46)		–	–	–	0.711

a *Independent samples t-test*.

b *Chi-squared analysis*.

*SD, standard deviation; FMA_UE, Upper Extremity Fugl-Meyer Assessment*.

*– Unavailable*;

* *Follow-up assessment was performed for 12 left-sided and 20 right-sided stroke patients*.

This study was approved by the Institutional Review Board of the Second Affiliated Hospital and Yuying Children's Hospital of Wenzhou Medical University, China (Approval number: Clinical Scientific Research Ethical Review No. 2017LCKY-09) and all the participants provided written informed consent. All the research procedures were conducted in accordance with the Declaration of Helsinki.

### Clinical Assessment and Rehabilitation

Clinical measurement related to motion performance was assessed. FMA_UE was used to measure upper limb movement, a highly recommended clinical tool for evaluating changes in motor impairment after stroke (range: 0–66) (24, 25). The upper extremity motor domain includes movement, coordination, and reflex action of the shoulder, elbow, forearm, wrist, and hand (24) and each item has a 3-point ordinal scale (0-cannot perform, 1-performs partially, and 2-performs fully) (25). All the patients received two assessments of FMA_UE at the following time points: immediately following enrollment (baseline FMA_UE) and a follow-up 15 weeks after enrollment (follow-up FMA_UE). To evaluate the extent to which upper extremity was recovered postrehabilitation, FMA_UE recovery ratio was defined as


$$\begin{array}{l} \text{rFMA\_UE}=\frac{\text{follow}-\text{up FMA\_UE }-\text{ baseline FMA\_UE }}{\text{baseline FMA\_UE}} \end{array}$$

and referred to as rFMA_UE. The positive and negative signs of rFMA_UE indicated improvement and deterioration of the upper extremity, respectively.

The FMA_UE assessments were evaluated by the same physician and collected on an electronic tablet device using Research Electronic Data Capture (REDCap) (26), a secure, convenient, and efficient web application for capturing electronic survey data.

All the patients received standard rehabilitation treatments after enrollment. The treatment program consists of physical and occupational therapy, including grips and finger movements, gross movement, stretching, and training in daily life activities.

### Magnetic Resonance Imaging Data Acquisition

Subjects were scanned on a 3 Tesla GE-Discovery 750 scanner with the following parameters: for anatomical T1-MRI data: TR/TE = 7.7/3.4 ms, flip angle = 12°, FOV = 256 × 256 mm, resolution = 256 × 256, slice per volume = 176, slice thickness = 1 mm; for fMRI data with odd interleave slice acquisition scheme: TE/TR = 30/2,500 ms, voxel size = 3.4375 × 3.4375 × 3.5 mm^3^, in-plane resolution = 64 × 64, number of volumes = 230, and flip angle = 90°.

### FMRI Data Preprocessing and Registration

A scrubbing-based preprocessing method (27) was applied to all rs-fMRI data with the following steps: discard first four volumes; motion correction; slice-time correction; intensity normalization; high-pass temporal filtering (0.008 Hz) for correcting low-frequency signal drift; regression of six motion vectors, cerebrospinal fluid (CSF) signal-averaged overall voxels of eroded ventricle region, averaged white matter (WM) signal, and averaged global signal of whole brain; motion-volume censoring by detecting volumes with frame-wise displacement (FD) larger than 0.5 mm, derivative variance root mean square (DVARS) after *Z* normalization larger than 2.3, and SD after *Z* normalization larger than 2.3, and scrubbing above detected (number of volume = *i*) and adjacent four volumes (*i–*2, *i–*1, *i, i*+1, *i*+2) (27, 28). FD is a measure of head motion from one volume to the next, and is calculated as the sum of absolute value of three translational displacements in *x, y, z* axis and three rotational displacements in *pitch, yaw*, and *roll* (units of radians), which were multiplied 50 to convert to similar units to translational displacements (27). DVARS is a measure of the change in volume intensity within a predefined gray matter (GM) mask from one volume to the next, calculated as the root mean square of the backward differentiated volumes; SD is a measure of deviation of volume intensity within the predefined GM mask. Because we were interested in the low-frequency fluctuations of resting-state fMRI signal, the aforementioned scrubbed time series were band-pass filtered (0.008–0.1 Hz) by applying a 4th-order Butterworth filter.

All the preprocessed fMRI data were registered to MNI152 2 mm template using a two-step procedure [ref. https://www.fmrib.ox.ac.uk/datasets/techrep/tr07ja2/tr07ja2.pdf]: the mean of preprocessed fMRI data were registered with a seven degrees of freedom affine transformation to its corresponding anatomical brain (FLIRT). Transformation parameters were also computed by non-linearly registering the individual brain to the MNI152 template (FNIRT). Combining the two transformations by matrix multiplication yielded transformation parameters normalizing fMRI data to standard space. All the final registered images were manually examined. Because the network analysis was voxel-wise-based, all registered images were down-sampled with linear interpolation to 6 × 6 × 6 mm^3^ for decreasing computational intensity.

We used 157 healthy subjects to generate an in-house 6 × 6 × 6 gray matter template. The procedure was briefly introduced, for each subject, first skull-stripped T1-brain image was segmented into gray matter (GM), white matter (WM), and cerebrospinal fluid (CSF) (FSL/FAST); second, GM was nonlinearly registered to MNI152_222 template (FSL/FNIRT); third, the 157 registered GMs were merged and averaged across subjects; the averaged GM was threshholded by 50% probability (the voxels were excluded if less than 79 subjects were covered). Final, the thresholded GM was downsampled to 6 × 6 × 6.

### Quality Control

After preprocessing, fMRI data were assessed for excessive motion. The number of censored motion volumes after preprocessing reflects the extent of motion of a subject during scanning. Any subject with <120 remaining volumes was excluded, which guaranteed a minimum of 5-min-scanning images for FC analysis (29).

### Brain Functional Connectivity Network and Global Graph Properties

Brain FC network that covered whole gray matter was constructed before global graph properties were computed. First, blood oxygenation level-dependent (BOLD) signal was extracted from each gray matter voxel in registered 6 × 6 × 6 m^3^ rs-fMRI data. Following this, a correlation matrix was generated by calculating voxel-based pairwise Pearson correlation coefficients of BOLD signals. Fisher's *z* transformation was applied to convert the Pearson correlation coefficient. To normalize the variation of strength of brain FC across individuals, a link density, the percentage of links with respect to the maximum number of possible links, was predetermined, which corresponds to a threshold (30, 31). In this study, 10 link densities (from 1 to 10%) were applied. Consequently, an indirectly connected brain FC network was generated after the correlation matrix was binarized by the subject-dependent threshold.

For each predetermined link density and each subject, five voxel-level graph properties were, respectively, computed using the brain connectivity toolbox (BCT) (32): “degree”—a measure of network hubness, “clustering coefficient”—a measure of network segregation, “betweenness centrality”—a measure of within-network communication, “efficiency”—a measure of network integration, and “participation coefficient”—a measure of diversity within a network.

### Graph Topological Disruption Index (K_D_) Comparison and Association With FMA_UE

K_D_ is used to measure the extent of brain functional reorganization, describing the topological changes of individual brain networks with respect to referential network topology (31), and indicating brain network property changes of a subject in some regions with the opposite trend in other regions (30, 33, 34). In this study, using brain of HCs as a normative reference, five graph topological disruption indices were computed: degree (D), betweenness centrality (BC), clustering coefficient (CC), efficiency (E), and participation coefficient (PC), and referred as K_D__D, K_D__BC, K_D__CC, K_D__E, and K_D__PC, respectively. For each graph topological disruption index, a repeated measures analysis of covariance (ANCOVA) with age and gender as covariates of no interest across all link density was performed to determine if there exists a significant difference between stroke patients (left-sided and right-sided, independently) and HCs. In addition, a Spearman correlation with age and gender as covariates was applied to examine if there exists a significant association between graph topological disruption indices and FMA_UE.

### Local Degree Comparison and Association With FMA_UE

Because the five graph topological disruption indices are significantly correlated (35) and degree is an important marker of network development and resilience (32), degree was used for exploring brain FC locally. Degree map was generated from the brain FC network by counting the number of functional links on the gray matter at each voxel. To decrease the effect of motion on local FC (27), the links within two adjacent voxels were excluded. To identify voxel-wise degree differences between patients (life-sided or right-sided) and HCs, the script of *randomize* in FSL is performed to generate 5,000 permutations of the data while controlling for age and gender as confounds; family-wise correction (corrected *p* = 0.05) was applied with threshold-free cluster enhancement (TFCE) (36). In addition, for each significant cluster found earlier, we further analyzed the association between the average degree count extracted from the cluster and FMA_UE. Using the script of *fslmeants* in FSL, the mean degree count across patient groups within each significant cluster was extracted, and Spearman correlation analysis was applied to examine if there exists a significant association between degree count and FMA_UE.

### Functional Connectivity Between Right Lateral Occipital Cortex (R LOC) and Other Nodes in the Subgroup of Right-Sided Stroke Patients

After R LOC was discovered as the only region in which there existed significant association between local degree count and FMA_UE in the right-sided stroke patient subgroup, to explore which regions R LOC connected to and if these regions were significantly different between right-sided stroke patients and their age- and gender-matched HCs, R LOC was used as a region of interest (ROI) to generate ROI-based degree maps for both right-sided stroke patients and HCs. To identify the difference of ROI-based degree map between right-sided stroke patients and HCs, the script of *randomize* in FSL is performed to generate 5,000 permutations of the data while controlling for age and gender as confounds; family-wise correction (corrected *p* = 0.05) was applied with threshold-free cluster enhancement (TFCE) (36).

### Software

All statistical analyses were performed using MATLAB 2016a, SPSS 23 (IBM Corp. in Armonk, NY), and FSL (www.fmrib.ox.ac.uk/fsl). Brain schemas of ROI and FC network were visualized on a surface rendering of a human brain atlas with the BrainNet Viewer (http://www.nitrc.org/projects/bnv/) (37).

## Results

### Comparisons of Demographics Between Stroke Patients and HCs and Clinical Characteristics of the Patients

As shown in Table 1, all 57 stroke patients were diagnosed with ischemic stroke. Of the 57 patients, 29 stroke patients had a left-sided stroke and the other 28 had a right-sided stroke, there were no statistical differences in age (*p* = 0.192, 0.770) or gender (*p* = 0.143, 0.605) between patients and their corresponding HCs. A total of 17 left- and 8 right-sided stroke patients did not participate in the follow-up assessment of FMA_UE.

### Significantly Disrupted Global Brain Connectivity in Stroke Patients

**Figure 1:**
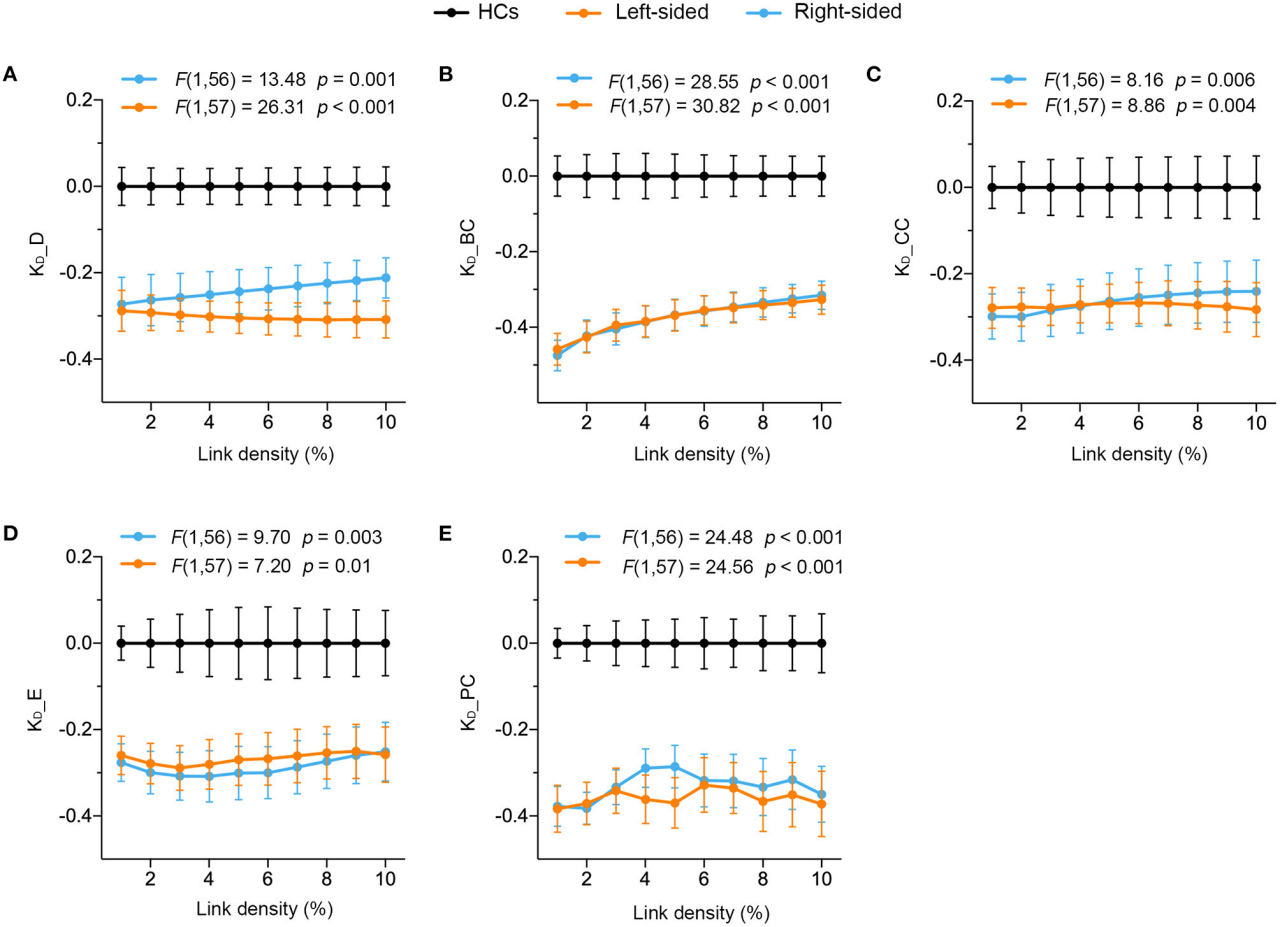
Significantly altered global connectivity was observed in left- and right-sided stroke patients. A repeated measure ANCOVA with age and gender as covariates of no interest determined that **(A)** K_D__D [*F*_(1, 57)_ = 26.31, *p* < 0.001], **(B)** K_D__BC [*F*_(1, 57)_ = 30.82, *p* < 0.001], **(C)** K_D__CC [*F*_(1, 57)_ = 8.86, *p* = 0.004], **(D)** K_D__E [*F*_(1, 57)_ = 7.20, *p* = 0.01] and **(E)** K_D__PC [*F*_(1, 57)_ = 24.56, *p* < 0.001] of left-sided stroke patients (orange) and **(A)** K_D__D [*F*_(1, 56)_ = 13.48, *p* = 0.001], **(B)** K_D__BC [*F*_(1, 56)_ = 28.55, *p* < 0.001], **(C)** K_D__CC [F_(1, 56)_ = 8.16, *p* = 0.006], **(D)** K_D__E [*F*_(1, 56)_ = 9.70, *p* = 0.003] and **(E)** K_D__PC [*F*_(1, 56)_ = 24.48, *p* < 0.001] of right-sided stroke patients (cyan) significantly decreased compared to HCs across all link densities. Data plotted as mean ± SE.

A repeated measures ANCOVA with age and gender as covariates of no interest determined that significant global altered connectivity was observed in stroke patients. All five graph topological disruption indexes of the left-sided stroke patients statistically significantly decreased compared to HCs across all link densities (from 1 to 10%) (see Figure 1, orange). The same significant decrease was also observed in the right-sided stroke patients (see Figure 1, cyan). Therefore, we may conclude that global brain connectivity in both right- and left-sided stroke patients was significantly disrupted compared with that in HCs. However, across all the link densities, these five disruption indexes were not significantly associated with baseline FMA_UE for both right- and left-sided stroke patients (see Table 2), indicating that none of the global disruption indexes are promising predictors of motor impairment after stroke.

**Table 2:** No significant correlations between the 5 disruption indexes in 2 subgroups and baseline FMA_UE at link density = 10%.

	**K_**D**__D**	**K_**D**__BC**	**K_**D**__CC**	**K_**D**__E**	**K_**D**__PC**
**Left_sided**					
*R*	−0.091	0.042	0.032	−0.088	0.228
*p*-value	0.651	0.835	0.876	0.661	0.254
**Right_sided**					
*R*	0.202	−0.035	0.111	0.130	−0.056
*p*-value	0.321	0.867	0.588	0.525	0.785

*K_D__D, disruption index of degree; K_D__BC, of betweenness centrality; K_D__CC, of clustering coefficient; K_D__E, of efficiency; K_D__PC, of participation coefficient*.

### Significantly Altered Local Connectivity in Stroke Patients

**Figure 2:**
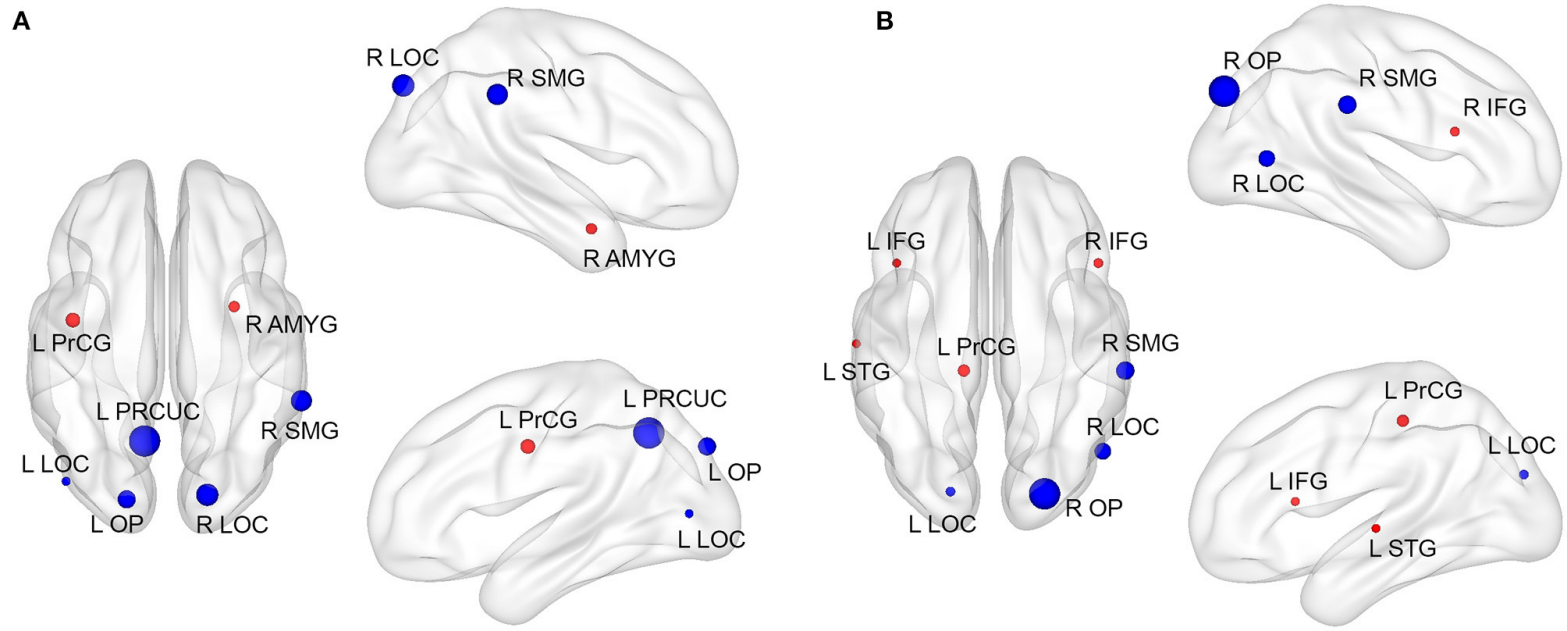
Significant local altered connectivity was observed in left- and right-sided stroke patients. Compared with HCs, the location and cluster size of regions with increased (red) and decreased (blue) local degree was depicted in **(A)** for the left-sided stroke patients, **(B)** for right-sided stroke patients. Red and blue nodes represented where nodal degree of stroke patients was greater and less than HCs, respectively. The size of the node represents cluster size. Significant level was set at *p* < 0.05 after multiple comparison corrections.

In addition to the global connectivity alteration observed in stroke patients, we also found altered local connectivity measured by degree in stroke patients. Compared with HCs, patients with left-sided stroke exhibited significantly increased nodal degree in the left precentral gyrus (L PrCG) and right amygdala (R AMYG), and the decreased nodal degree in the left precuneous cortex (L PRCUC), right supramarginal gyrus (R SMG), left occipital pole (LOP), right lateral occipital cortex (R LOC), and L LOC (Table 3A; Figure 2A). Patients with right-side stroke showed significantly increased nodal degree in the L PrCG, right inferior frontal gyrus (R IFG), left superior temporal gyrus (L STG) and L IFG, and the decreased nodal degree in the right occipital pole (R OP), R SMG, R LOC, and L LOC (Table 3B; Figure 2B).

**Table 3:** Brain regions with significant local degree differences between stroke patients and HCs.

**Brain regions**	**MNI coordinates**			**Cluster size (mm^**3**^)**	***t*_(59)_-value**	***p*-value**
	**X**	**Y**	**Z**			
**(A) Brain regions with significant local degree differences between left-sided patients and HCs at 10% link density**						
**Stroke Patients** **>** **HCs**						
Left precentral gyrus	−42	−6	36	1,296	4.12	**<** **0.001**
Right amygdala	30	0	−24	864	4.17	**<** **0.001**
**Stroke Patients** **<** **HCs**						
Left precuneous cortex	0	−60	42	9,504	4.25	**<** **0.001**
Right lateral occipital cortex^ 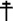 ^	18	−84	42	3,240	4.77	**<** **0.001**
Right supramarginal gyrus^ 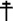 ^	60	−42	36	2,808	4.42	**<** **0.001**
Left occipital pole^ 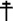 ^	−18	−90	36	1,944	4.29	**<** **0.001**
Right lateral occipital cortex^ 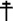 ^	60	−66	0	1,080	3.90	**<** **0.001**
Left lateral occipital cortex	−48	−78	6	648	3.64	**<** **0.001**
*t*_(59)_: 59 represents degree of freedom. ^ 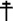 ^: overlapped region with that from right-sided patients						
**(B) Brain regions with significant local degree differences between right-sided patients and HCs at 10% link density**						
**Stroke Patients** **>** **HCs**						
Left precentral gyrus	−12	−30	48	1,512	4.77	**<0.001**
Right inferior frontal gyrus	48	18	18	864	3.19	**0.002**
Left superior temporal gyrus	−60	−18	0	648	4.38	**<0.001**
Left inferior frontal gyrus	−42	18	12	648	3.95	**<0.001**
**Stroke Patients** **<** **HCs**						
Right occipital pole^ 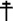 ^	24	−90	36	6,264	4.49	**<0.001**
Right supramarginal gyrus^ 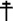 ^	66	−30	30	3,024	4.09	**<0.001**
Right lateral occipital cortex^ 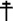 ^	60	−66	6	2,592	4.65	**<0.001**
Left lateral occipital cortex^ 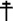 ^	−18	−84	24	864	3.30	**0.002**

*t_(58)_, 58 represents degree of freedom; ^
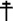
^Overlapped region with that from left-sided patients. Bold values of p-value represents statistically significance (p < 0.05)*.

### Degree in R LOC Was Associated With Baseline FMA_UE and Predicted the Extent of Motion Recovery in the Right-Sided Stroke Patients

**Figure 3:**
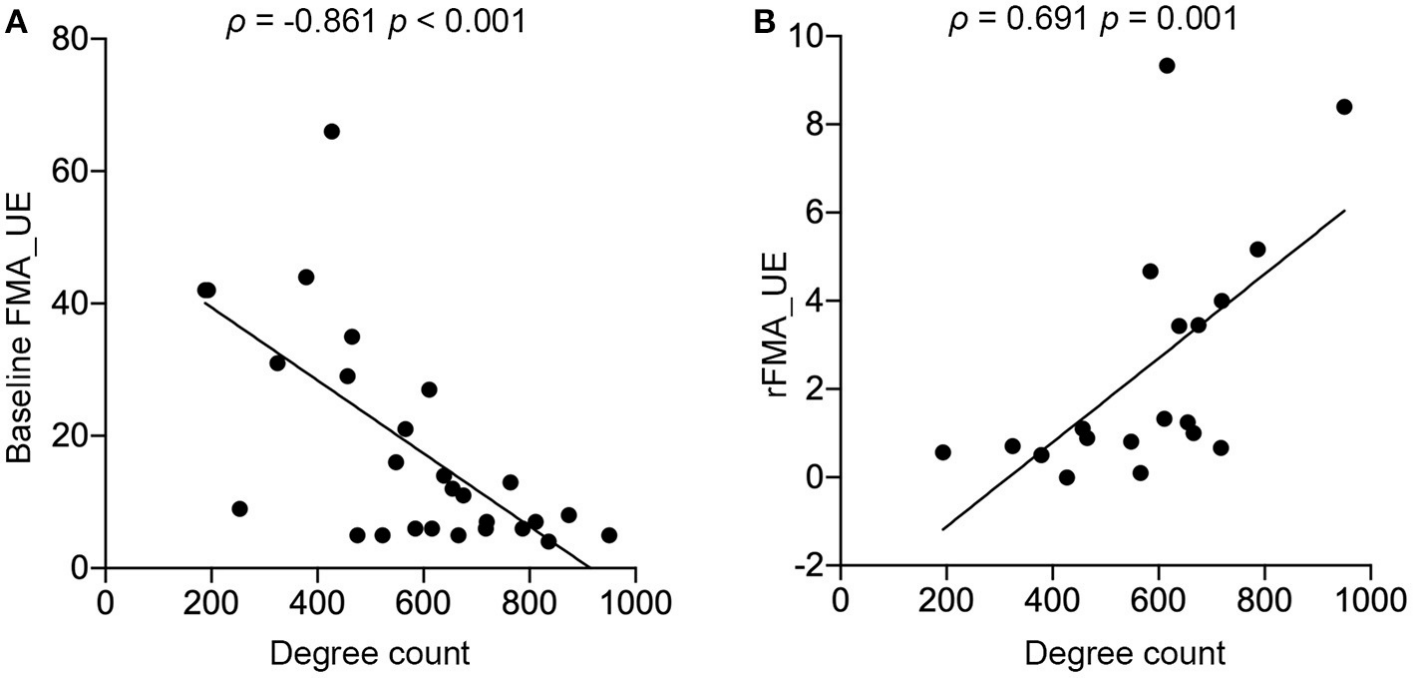
Significant associations between degree and upper extremity motor performance were discovered in R LOC in the right-sided stroke patients. **(A)** Baseline FMA_UE scores significantly correlated to local degree in R LOC; **(B)** rFMA_UE significantly correlated to local degree in R LOC.

We further analyzed the associations between the local degree count extracted from significant brain regions (Table 2B; Figure 3) in both left- and right-sided stroke patients and their baseline FMA_UEs. Only R LOC in the group of the right-sided stroke patient was significant after a Bonferroni correction (*p*_Bonferroni_ = 0.006) (ρ = −0.861, *p* < 0.001, Figure 3A). Moreover, the degree in R LOC was able to predict rFMA_UE, the extent of upper extremity recovery after rehabilitation (ρ = 0.691, *p* = 0.001, Figure 3B).

### ROI-Based FC Analysis Revealed Regions With Significantly Different FC in Right-Sided Stroke Patients

**Figure 4:**
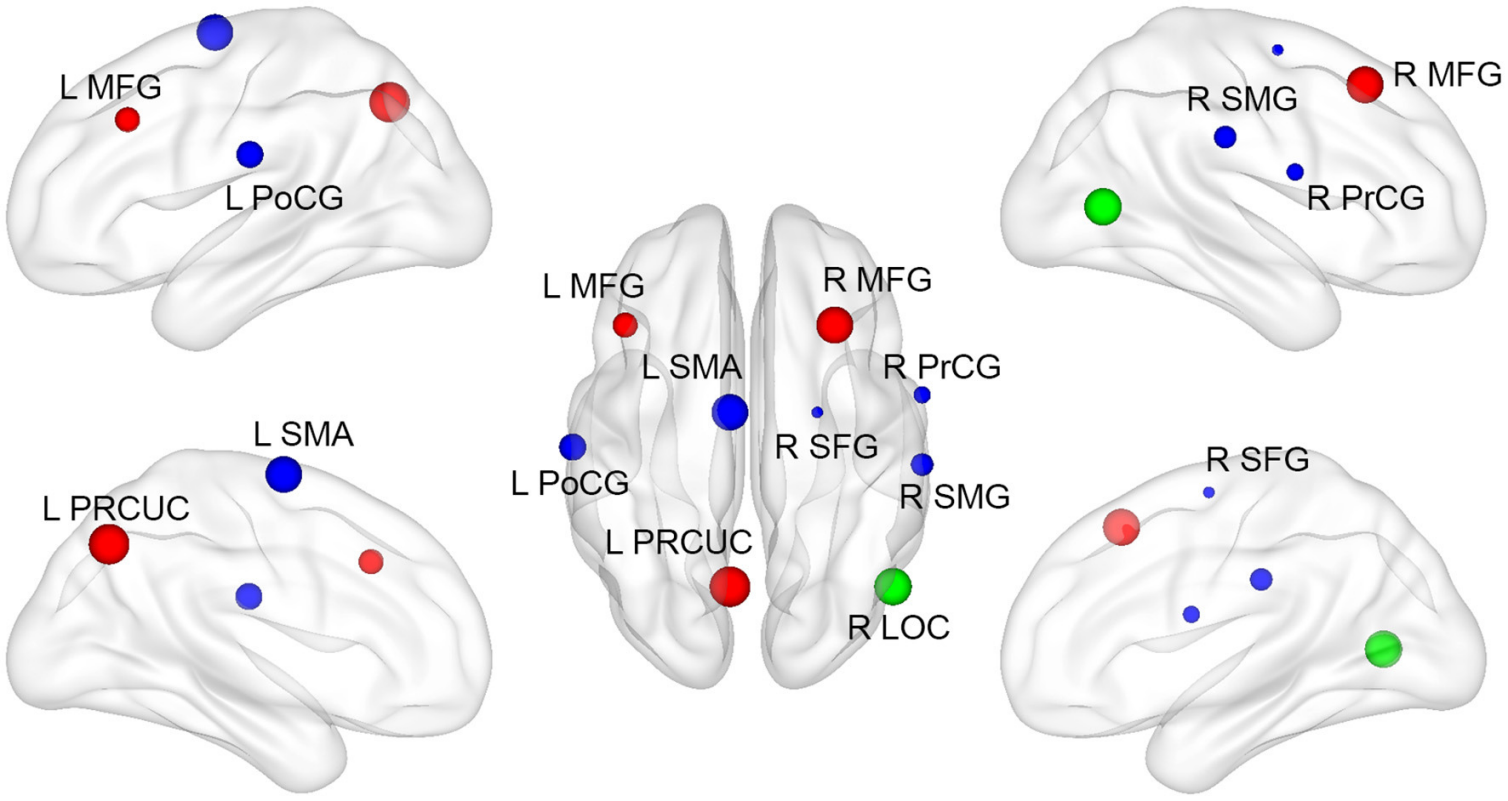
Regions with significantly different ROI-based functional connectivity in right-sided stroke patients. Compared to HCs, the location and cluster size of regions with significantly different R LOC-based functional connectivity. Red and blue nodes represent those regions of which functional connections were greater and less than HCs, respectively. The green node represents the seed ROI (R LOC).

By using R LOC as an ROI, the region in which local degree alteration was associated with motor performance, an ROI-based FC analysis revealed regions with significantly different FC in right-sided stroke patients compared with HCs. The regions with increased degree connectivity to R LOC were L PRCUC, right middle frontal gyrus (R MFG), and left middle frontal gyrus (L MFG). The regions with decreased degree connectivity to R LOC are the left supplementary motor cortex (L SMA), left postcentral gyrus (L PoCG), R SMG, R PrCG, and right superior frontal gyrus (R SFG) (Figure 4). The detailed MNI coordinates and the cluster size are listed in Table 4.

**Table 4:** Brain regions with significant differences in R LOC functional connectivity between right-sided stroke patients and HCs.

	**MNI coordinates**			**Cluster size (mm^**3**^)**	***t*_(58)_-value**	***p*-value**
	**X**	**Y**	**Z**			
**Stroke Patients** **>** **HCs**						
Left precuneus	−6	−66	42	8,640	4.19	**<0.001**
Right middle frontal gyrus	30	24	48	6,264	5.64	**<0.001**
Left middle frontal gyrus	−42	24	36	2,160	3.57	**<0.001**
**Stroke Patients** **<** **HCs**						
Left supplementary motor cortex	−6	−6	66	6,048	3.53	**<0.001**
Left postcentral gyrus	−60	−18	24	2,592	4.51	**<0.001**
Right supramarginal gyrus	66	−24	30	1,728	4.03	**<0.001**
Right precentral gyrus	60	0	18	1,080	4.07	**<0.001**
Right superior frontal gyrus	24	−6	60	648	3.30	**0.002**

*Bold values of p-value represents statistically significance (p < 0.05)*.

## Discussion

A key theoretical and clinical question of interest is the nature of any topological abnormalities in the brain network organization of stroke patients which might relate to their motor performance; assessing this would shed light on which aspects of normal brain network organization might be critical for upper limb motor recovery.

In this study, we applied graph-based theoretical approaches to rs-fMRI to analyze the brain functional connectome topology in subacute ischemic stroke compared with HCs. Our main findings are summarized as follows: (1) at the global level, using brain of HC as a normative reference, there exists significantly disrupted global brain connectivity in subacute stroke patients compared with HCs, but no correlation between global disruption indexes and clinical measurements was found; (2) at the local nodal level, by measuring degree, significant local degree differences between stroke patients and HCs were found, among which baseline FMA_UE and rFMA_UE were significantly correlated to the degree count in R LOC in right-sided stroke patients.

We found that whole-brain network structure of the stroke patients was altered, regardless of stroke site. Graph disruption indices derived from five network topological measurements, degree, clustering coefficient, participation coefficient, betweenness centrality and efficiency, which, respectively, represent network hubness, segregation, diversity, within-network communication, and integration, were significantly decreased compared with HCs across all predefined link densities in two subgroups. The results, in accord with findings of Termenon et al. (33) of significantly lower K_D_ in the contralesional hemispheres of brain networks of the patients, indicates that stroke-induced whole brain reorganization, suggesting that FC and other network properties vary in the certain regions at the subacute stage. The failure to observe any significant association between global hub disruption and FMA_UE across two subgroups reveals that although global hub disruption is a more reliable and sensitive metric than graph metrics to detect brain reorganization (33), it is not a biomarker to predict the motor dysfunction caused by stroke at the subacute stage, which implies that the clinical measurement reflecting motor dysfunction might only be related to brain reorganization within brain network(s) rather than whole brain reorganization.

We found that local-brain network structure of stroke patients was altered in terms of degree compared with HCs. From data shown in Figure 2, we conclude that the local altered regions were not identical across two subgroups. In general, the increased degree was mainly located in the temporal and frontal lobes, decreased in the occipital lobe, and altered regions were located not only in the hemisphere of the lesion, but also in the contralesional hemisphere. These findings might reflect the overall impact of local lesions on the long-distance functional connective area and indicate that the visual, motor, emotional, language processing, and cognitive processes of patients with subacute ischemic stroke were damaged or at least degraded.

The right-sided stroke patients, where the degree extracted from degree-altered regions was significantly correlated to the upper extremity motor function, showed significantly decreased degree in the occipital cortex and right supramarginal gurus, and increased degree mainly in the L PrCG, R IFG, L STG, and L IFG (see Figure 2B; Table 3B). The observation of decrement of degree in the occipital cortex is consistent with findings in patients with transient ischemic attack (TIA) (38) and ischemic stroke patients (39), as this region is a part of the visual dorsal stream and is involved in object localization (40). The right-sided stroke patients with more severe paralysis (lower FMA_UE) had more FC in the R-LOC (Figure 3A), which could be attributed to functional compensation after the onset of stroke (41). Similarly, for the regions with increased degree, previous studies have demonstrated that altered organization of connectivity in the middle frontal gyrus in patients with subcortical stroke reflected the role of the frontal lobe in higher-order movement planning (42, 43), as the frontal lobe is associated with the multiple forms of olfaction and higher-order cognition, including working memory, inhibitory control, conflict monitoring, and shifting between rule sets (44), and that the L PrCG is in the sensorimotor network (SMN), a network with multifunctional characteristics and vulnerable not only to neurodegenerative diseases, but also to cerebrovascular diseases (45, 46). The compensatory mechanism for motor impairment might contribute to this degree increment. However, we did not observe the same associations in the left-sided stroke patients. One of the possible reasons might be that left- and right-side stroke patients have significantly different topological properties (47, 48). Left-lateralization in the language process (49) and the left hemisphere being the dominant hemisphere of right-handers might result in the left-side stroke patients having more complex FC changes poststroke.

Our finding that the degree extracted from the R LOC region was significantly associated with upper extremity motor recovery [i.e., right-sided stroke patients with more severe paralysis had more potential to benefit from the compensatory mechanism, leading to a higher chance of FC in R LOC after 15 weeks rehabilitation (Figure 3B)], assists our understanding of the importance of eyehand coordination in the rehabilitation of upper limb motor function. The R LOC is part of the visual cortex (40) that connects with the amygdala, hippocampus, and *via* intrathalamic pathways with mediofrontal areas (50). The R LOC also receives top–down modulation from frontal and parietal areas in relation to visual attention (51). Previous studies indicate visual feedback can enhance motor function (52, 53) so that during rehabilitation, dependence on visual feedback increases (54, 55) and viewing motion stimuli leads to activity increases in regions of the extrastriate visual cortex (56). Moreover, multimodal fMRI experiments in (57) revealed that passive touch significantly activated the object selective parts of LOC and that the coupling was specific to hand and shoulder stimulation, suggesting that LOC is functionally connected to the hand area of primary somatosensory. While our data cannot provide causality direction between R LOC and upper extremity motor recovery, thereby preventing us from identifying it as a sure brain region to target for treatment, together, these findings, and the previous finding point to the R LOC as an important region to further probe for intervention methods of poststroke motor rehabilitation.

By virtue of an R LOC-based FC analysis, we identified the regions that connected to R LOC and the degree extracted from which was significantly increased compared to HCs at the subacute stage poststroke, left PRCUC and left and right MFG, and the regions with decreased connectivity, L SMA, L PoCG, R SMG, R PrCG, and R SFG. These regions correlated with the motor recovery after stroke (20, 58, 59). Combined with the discovery that the more the degree was extracted from the R LOC, the better motor function recovery the patient demonstrated, these widespread regions in both hemispheres might provide subacute stroke patients with a motor compensation mechanism after the onset of stroke.

There are limitations in this study. First, in our study, 74 and 54% of patients had hypertension, and diabetes, respectively, so variances in brain structure and function caused by hypertension (60), diabetes (61), and stroke are difficult to delineate. In the future, we will have a cross-sectional study on the differences between groups of stroke patients with and without hypertension and diabetes. Second, participants were not divided into groups according to the severity of motor dysfunction. Third, our sample size of 28 right-sided stroke patients is relatively small; future work will include a larger cohort of patients; and focus on the analysis of longitudinal changes in brain regions critical to stroke outcomes captured through brain functional topological properties. Fourth, we did not collect fMRI data during the follow-up period, and thus cannot detect how brain networks reorganize as stroke continues to evolve. Fifth, we did not collect language-related measurements, which prevented us from further investigating why left- and right-sided stroke patients had different associations between the degree extracted from R LOC and the upper extremity motor recovery.

## Conclusions

In conclusion, the five global graph topological disruption indices of stroke patients were significantly decreased in two subgroups compared with HCs. Correlation analysis revealed that these global disruption indices were not significantly associated with FMA_UE. Local brain FC in terms of degree was explored and it was found that local brain network structure of the stroke patients was altered and significant associations between region degree and upper extremity motor functional recovery were observed in R LOC in the subgroup of right-sided stroke patients. These findings suggest that the topological properties of functional brain networks may provide prognostic value for motor functional recovery after stroke.

## Data Availability Statement

The original contributions presented in the study are included in the article/supplementary material, further inquiries can be directed to the corresponding authors.

## Ethics Statement

The studies involving human participants were reviewed and approved by Institutional Review Board of the Second Affiliated Hospital and Yuying Children's Hospital of Wenzhou Medical University. The patients/participants provided their written informed consent to participate in this study.

## Author Contributions

SJ, LH, and QH contributed to conception and study design. WT, DL, YC, YJ, and LF contributed to acquisition of data. QH, LH, SH, and BW analyzed functional images and statistical analysis. LH and QH drafted and edited the manuscript. All authors approved the final manuscript version to be published.

## Funding

QH received a grant from the Wenzhou Bureau of Science and Technology (No. Y20190042). SJ received a grant from the Medical Health Science and Technology Project of Zhejiang Provincial Health Commission (No. 2017198456).

## Conflict of Interest

The authors declare that the research was conducted in the absence of any commercial or financial relationships that could be construed as a potential conflict of interest.

## Publisher's Note

All claims expressed in this article are solely those of the authors and do not necessarily represent those of their affiliated organizations, or those of the publisher, the editors and the reviewers. Any product that may be evaluated in this article, or claim that may be made by its manufacturer, is not guaranteed or endorsed by the publisher.
